# Fatigue in Parkinson’s disease: report from a mutidisciplinary symposium

**DOI:** 10.1038/npjparkd.2015.25

**Published:** 2016-01-14

**Authors:** Joseph H Friedman, James C Beck, Kelvin L Chou, Gracia Clark, Christopher P Fagundes, Christopher G Goetz, Karen Herlofson, Benzi Kluger, Lauren B Krupp, Anthony E Lang, Jao-Shin Lou, Laura Marsh, Anne Newbould, Daniel Weintraub

**Affiliations:** 1 Movement Disorders Program, Butler Hospital, Province, RI, USA; 2 Department of Neurology, Alpert Medical School of Brown University, Providence, RI, USA; 3 Parkinson’s Disease Foundation, New York, NY, USA; 4 Department of Neurology, University of Michigan Health System, Ann Arbor, MI, USA; 5 Department of Neurosurgery, University of Michigan Health System, Ann Arbor, MI, USA; 6 Department of Psychology, Rice University, M.D. Anderson Cancer Center, Houston, TX, USA; 7 Department of Health Disparities, Houston, TX, USA; 8 Department of Neurological Sciences, Rush University Medical Center, Chicago, IL, USA; 9 Department of Neurology, Sorlandet Hospital, Arendal, Norway; 10 Department of Neurology, University of Colorado Anschutz Campus, Aurora, CO, USA; 11 Department of Neurology, Stony Brook University Medical Center, Stony Brook, NY, USA; 12 Toronto Western Hospital, Morton and Gloria Shulman Movement Disorders Clinic and Edmond J Safra Program in Parkinson’s Disease, Toronto, ON, Canada; 13 University of North Dakota School of Medicine and Health Services, Sanford Health, Grand Forks, ND, USA; 14 Department of Psychiatry, Baylor College of Medicine, Mental Health Care Line, Michael E. DeBakey Veterans Affairs Medical Center, Houston, TX, USA; 15 Department of Neurology, Baylor College of Medicine, Mental Health Care Line, Michael E. DeBakey Veterans Affairs Medical Center, Houston, TX, USA; 16 Department of Psychiatry, Perelman School of Medicine, University of Pennsylvania, Philadelphia, PA, USA

## Abstract

Fatigue is a severe problem for many people living with Parkinson’s disease (PD). Best estimates suggest that more than 50% of patients experience this debilitating symptom. Little is known about its etiology or treatment, making the understanding of fatigue a true unmet need. As part of the Parkinson’s Disease Foundation Community Choice Research Program, patients, caregivers, and scientists attended a symposium on fatigue on 16 and 17 October 2014. We present a summary of that meeting, reviewing what is known about the diagnosis and treatment of fatigue, its physiology, and what we might learn from multiple sclerosis (MS), depression, and cancer—disorders in which fatigue figures prominently too. We conclude with focused recommendations to enhance our understanding and treatment of this prominent problem in PD.

## Introduction

Fatigue is a common symptom in almost all medical, neurologic, and psychiatric disorders as well as in the otherwise healthy general population.^1^ We considered fatigue a subjective symptom, different from muscle fatigue, which is a physiologically defined entity. There are a number of definitions used to describe subjective fatigue,^2^ and all focus on the concept of an abnormal and excessive lack of energy that interferes with normal function. Although the recognition of the importance of fatigue in Parkinson’s disease (PD) is relatively new, it should be noted that Parkinson-related fatigue had been recognized in the nineteenth century by some of the major clinical neurologists of their time.^3^

## Defining fatigue

There are several difficulties in defining fatigue. Fatigue may occur in more than one domain^4,5^ and different scales include different domains. Defining the point at which normal fatigue crosses into pathological fatigue is also challenging. Operationally, fatigue becomes pathological when it is abnormally severe, restricts activities independently of other constraints induced by PD, and is associated with psychological distress; however, it remains difficult to define an exact diagnostic threshold.^6^ Fatigue may be associated or due to other behavioral disorders such as depression, anxiety, and apathy, which are themselves intrinsic to PD.^7^ In addition, there may be confusion between fatigue, a feeling present at rest, and fatigability, a problem induced by activity.^5^ Although PD fatigue has traditionally been considered a symptom, it may be better considered as a syndrome. Therefore, we propose to define PD fatigue as *a sense of exhaustion unexplained by drug effects, other medical, or psychiatric disorders, present for a defined period, and associated with other fatigue-related symptoms, such as reduced motivation and nonrestorative rest, or constraints on activities.* ^6^ Such a syndromic definition may provide several benefits for clinical research by improving comparability across studies but might also inhibit recruitment if the definition is too narrow or inflexible. However, this definition remains quite subjective, and until objective measures are discovered, which reflect the sensation of exhaustion, we will continue to be limited in our ability to understand the pathophysiology of this disorder.

## Clinical review of fatigue in PD

### Motor fatigue

Most studies of PD fatigue have focused on physical, or motor, fatigue. Case–control studies^8,9^ and community-based studies^10^ show fatigue to be both common and severe, with the prevalence ranging between 33 and 70%.^11^ This broad range probably reflects different patient populations and different evaluation methodologies. This prevalence makes it one of the most common nonmotor PD problems,^12^ even in the early stages of the disease. It is clearly a ''pre-motor'' feature as well, appearing well before motor symptoms.^13,14^ A UK study spanning 16 years, involving 8,166 patients with PD and 46,755 controls, found that the presence of fatigue 5 years before the diagnosis of PD increased the relative risk of developing the disease to 1.56 (ref. 15).

Patients describe their fatigue as different from what they had experienced previously.^16^ Unlike fatigue in the general population, PD fatigue often improves with exercise.^16,17^ Two studies evaluating fatigue in newly diagnosed, untreated patients reported it to be a clinically relevant problem^3,6,10,14^ even when motor symptoms were minimal. Over 50% of patients consider fatigue among their three most disabling symptoms.^8^ In spite of this prominent concern by patients, fatigue is often not recognized by the treating neurologist.^18^

The evolution of fatigue has not been well studied. Limited data indicate that, once present, fatigue typically persists and usually worsens over time.^19,20^

Studies of PD-related fatigue are discordant regarding the correlation between fatigue and motor symptom severity, disease stage, or duration.^2,14^ These conflicting results may be explained by different populations as well as different assessment tools. Multiple, but not all, studies in PD show a significant correlation between depression and fatigue.^8^ Anxiety and apathy also correlate with fatigue^21,22^ but many fatigued patients are not depressed, anxious, or apathetic.^8^ Several studies suggest that cognitive performance, frontal lobe activity, and self-reported cognitive impairment are associated with fatigue.^23,24^ However, fatigability on cognitive tasks may not correlate with subjective fatigue.^25^ Similar to depression, fatigue is frequently associated with daytime sleepiness and sleep disorders, but can be distinguished from them by the absence of sleep being restorative, and may occur in patients with normal sleep patterns.^8,9,26^ Finally, objectively measured autonomic dysfunction has been associated with fatigue in PD.^27,28^

### Cognitive fatigue

Cognitive fatigue refers to the subjective experience of feeling weary or exhausted during an intellectually challenging task, along with a decreased capacity to initiate or sustain cognitively challenging activities. Cognitive fatigue is sometimes referred to as ''central fatigue.'' Patients have reduced attention and may have deficits on formal neuropsychological testing in cognitive areas of memory, learning, attention, and information process.^29^ A limited number of studies have examined cognitive fatigue in PD.^30^ In some patients, cognitive performance is unimpaired; however, the increased mental effort required to maintain a normal level of performance results in fatigue and distress. Conversely, cognitive impairment, especially executive dysfunction, may be associated with cognitive fatigue, as the patient is less able to maintain concentration or think about complex matters. For example, a longitudinal decline in Mini-Mental State Examination score over 8 years was associated with a concurrent increase in fatigue symptoms.^19^ In addition, reduced performance on the Wisconsin Card Sorting Task, reduced frontal lobe perfusion, and depression were all correlated with fatigue.^23^ The development and experience of fatigue is also context-dependent; cognitive fatigue emerges as task complexity increases and attentional demands are greater. Even in healthy controls, increased task complexity during a walking exercise affects gait speed; however, the impact on physical fatigue and gait slowness is greater in patients with PD.^31^

## Insights into fatigue from other disorders

Although perceived fatigue is probably similar among different illnesses,^32^ there may be disease-specific differences. Both similarities and differences may provide insights into fatigue pathophysiology. Similarities in fatigue would be compatible with a hypothesis of shared mechanisms, and thus the possibility of similar interventions. The high prevalence of fatigue in medical and psychiatric disorders points either to some very generalized mechanisms or a limitation in the brain’s ability to distinguish perceptions. We limited our review of other disorders with prominent fatigue, but excluded chronic fatigue syndrome because of its frequent association with psychiatric disorders such as personality disorders and post-traumatic stress disorder that might confound physiological interpretations.

## Cancer

Fatigue has been most studied in oncology. Cancer specialists have developed a disease-specific definition for ''cancer-related fatigue'' as a syndrome that is included in the ICD 10.^6^ The goal of such a definition was to accelerate the ''development of a research agenda.'' ''Fatigue is the most common symptom experienced by adults and children with cancer'' (ibid). The criteria look much like typical criteria used in the Diagnostic and Statistical Manual V,^33^ the guide for psychiatric diagnoses, with a requirement for ''significant fatigue'' plus 5 symptoms from a list of 10, with symptoms present daily or almost daily for a 2-week period in the previous month.

Cancer, of course, is not a single disease. Many of the links found between certain cancers and fatigue have not been evaluated in other cancers. Cytokines, released during inflammation, have been correlated with fatigue in cancer.^34,35^

Hypothalamic–pituitary axis alterations have been implicated.^36^ Changes in circadian rhythm^37^ have been associated with fatigue as has a genetic predisposition.^36^ Psychosocial factors also have a high correlation with fatigue.^38^

## Multiple sclerosis

The estimated prevalence of fatigue in multiple sclerosis (MS) is ~80%. ''Chronic persistent fatigue'' is defined for MS as significant fatigue present for more than 50% of each day for a minimum of 6 weeks.^39^ There are some facets of fatigue that distinguish MS fatigue from fatigue associated with other disorders. Unlike PD, fatigue in MS does not decrease with exercise.^17^ Heat worsens fatigue in MS, an unusual but consistent observation compared with other disorders.^40^ This has not been studied in PD but anecdotal experience indicates that PD patients routinely prefer warm to cold climates, suggesting that heat is unlikely to worsen fatigue.

Fatigue severity in MS is linked to disease type (chronic progressive more likely to be affected), but, not clearly linked to disability or disease duration.^39^ It is closely related to concurrent depression, and the presence of depression or anxiety predicts the later occurrence of fatigue. Similarly, the presence of fatigue is a risk factor for later development of depression and anxiety. Sleep disorders are increased in MS subjects with fatigue. Lower levels of education are associated with worse fatigue. As in PD,^41^ the sensation of fatigue does not correlate with motor fatigue, the loss of strength with repeated effort (see below). Fatigue has been associated with a variety of gray matter changes in MS as well as functional magnetic resonance imaging changes during tasks.^40^ There may be associations between fatigue and neuroendocrine, autonomic, or neuroimmune markers.

## Depression

Although fatigue is one of the symptoms used to diagnose major depression in the general population,^33^ it is often unrelieved when depression improves.^42–44^ Thus, even when the fatigued PD patient has depression, treatment of the latter may not improve the former, thus implying that fatigue may not always simply result from depression.

The data on cancer and MS fatigue are instructive in terms of identifying areas of research that have not been adequately explored in PD. These areas include pain, socioeconomic and educational status, a variety of psychosocial measures, markers of inflammation and immune function, deconditioning, and autonomic dysfunction; none of these observations in other disorders have resulted in clear benefit to patients. A study comparing consecutive people with MS to those with PD, both groups attending similar academic subspecialty neurology clinics, found that the spectrum of symptoms endorsed in the Fatigue Severity Scale (FSS) were not identical.^17^ In addition, patients with PD generally reported that fatigue improved with exercise, whereas those with MS reported the opposite. Whether this counterintuitive observation about exercise is unique to PD fatigue remains to be seen.

## Clinical measures of fatigue in PD

A systematic critique of rating scales for diagnosis and severity of PD fatigue used pre-determined criteria for Recommended, Suggested, or Listed, depending on the quality of data.^45^ The FSS met the necessary criteria to be recommended for both diagnostic screening and severity measurement.^2,46^ The Multidimensional Fatigue Inventory (MFI)^4^ was designated as recommended for rating fatigue severity, and may be more sensitive to change with interventions than the FSS.^46^ For diagnostic screening only, two other scales were recommended: Functional Assessment of Chronic Illness Therapy—Fatigue Scale (FACIT-F)^47^ and Parkinson Fatigue Scale (PFS or PFS-16).^16^ Since the MDS review article, the Modified Fatigue Impact Scale has been validated in a study involving 100 PD patients.^48^ This scale involves evaluation of cognitive as well as physical and social functioning.

The above scales measure subjective fatigue in PD. Objective motor fatigue can also be measured in PD with performance changes during sustained muscle contractions, repetitive movements, or timed activities. PD studies generally show heightened motor fatigability, which does not typically correlate with subjective fatigue.^49^ Objective measures of cognitive fatigability focus on performance change during a continuous mental task or performance change in another target task after a cognitive challenge.^50,51^ Performance variability over time may be a sensitive metric of fatigability that correlates with fatigue complaints.^51^ A study using the Borg Perceived Exertion Rating^52^ measuring perceived effort documented heightened perception in PD in comparison with controls, despite similar levels of performance.^53^

### Physiology and biomarkers for fatigue in PD

The physiology underlying fatigue symptoms in PD is unknown, whereas much is known about the mechanisms of motor fatigability. The only published study of physiological differences between fatigued and nonfatigued PD patients found no measurable differences in oxygen utilization during exercise,^54^ but some studies have suggested that exercising improves fatigue.^15,17,55,56^ Whether fatigue fluctuates with motor fluctuations was looked at in one study.^57^ Subjects were assessed during their off, whereas all other studies used the subjects’ experience over a preceding time interval of weeks. Eighty-eight percent of their subjects were fatigued, and fatigue increased with motor ''off.'' Too few subjects were nonfatigued to determine how often fatigue occurred only during motor ''off.'' The physiological implications are unclear, as many other nonmotor symptoms also increased during the ''off'' period.

Increased motor fatigability in PD is associated with increased cortical excitability. Motor fatigability is defined as the rate of force deterioration in an exercise paradigm. Cortical excitability may be measured by transcranial magnetic stimulation applied to the motor cortex with the compound muscle action potentials recorded from the muscles of interest such as the abductor pollis brevis or the first dorsal interosseous muscle. Cortical excitability is increased when the motor-evoked potential amplitude increases while transcranial magnetic stimuli are held constant. The cortical excitability is markedly increased in PD at rest, and during intermittent exercise that correlates negatively with the severity of motor fatigue—the higher the cortical excitability, the less motor fatigability.^58^ The increase in cortical excitability is partially normalized by a small dose of levodopa that also reduces motor fatigability;^58,59^ however, neither cortical excitability nor motor fatigability correlated with subjective fatigue complaints. Thus, cortical excitability can serve as a biomarker for motor fatigability in PD but not for subjective fatigue.

## Biomarkers

Inflammatory markers such as C-reactive protein and cytokines are potential biomarkers for subjective fatigue. In a study comparing 87 PD subjects to 33 controls, several markers of inflammation were assessed in the cerebrospinal fluid and correlated with a variety of nonmotor symptoms. Elevated C-reactive protein and monocyte chemotactic protein-1 were significantly correlated with fatigue, but this association requires confirmation before being considered clinically relevant.^60^ How serum markers relate to brain changes in PD is speculative; however, they may relate to autonomic changes outside the central nervous system.

### Imaging of fatigue in PD

Motor symptoms in PD result from nigrostriatal dopaminergic denervation, but dopaminergic dysfunction does not appear to be related to fatigue in PD. In the ELLDOPA cohort, 49 levodopa-naive PD patients with fatigue had similar [^123^I]-β-CIT striatal dopamine transporter uptake as 82 PD patients without fatigue.^14^ Another study showed no difference in ^18^F-dopa uptake between 10 fatigued PD subjects and 10 nonfatigued PD subjects.^2,61^

Serotonin transporter uptake has been reported to be reduced in chronic fatigue syndrome,^3,61^ suggesting that nondopaminergic pathways may be involved in PD fatigue. Pavese *et al*.^62^ compared serotonergic transporter uptake (using the positron emission tomography (PET) ligand ^11^C-DASB) in seven PD subjects with fatigue and eight PD subjects without fatigue. Serotonin transporter binding in the caudate, putamen, ventral striatum, insula, and thalamus was decreased in the fatigued patients. The relationship of the cholinergic system to PD fatigue has not been investigated. The autonomic nervous system may also be involved in PD fatigue. One study found that pressor responses in norepinephrine and dobutamine infusion tests were greater and MIBG cardiac uptake was decreased in PD subjects with fatigue compared with those without fatigue.^4,27^

## Current treatment approaches

There are no evidence-based guidelines for treating fatigue in PD. A recent review^63^ found only 14 published randomized, controlled trials using validated fatigue measures in PD. No study of nonpharmacological treatments had showed benefit for fatigue. Only one study showed a clinically and statistically significant improvement with stimulants;^64^ however, when data from other stimulant trials were pooled, no effect was seen. Statistically but clinically insignificant benefits were found in *post hoc* analyses of rasagaline,^65^ and small benefits were seen for levodopa and other drugs when fatigue was one of several nonmotor symptoms assessed in randomized, placebo-controlled trials, where nonmotor symptoms were not the primary end points of the study. Nonpharmacologic approaches to treat cognitive fatigue include exercise, daily scheduling, planned rests, and pacing daily activities, similar to what has been tried in treating motor fatigue. The Table 1 summarizes interventions reported upon.

Given the limited evidence regarding treatment of PD fatigue,^66^ a commonsense approach to treat fatigue is recommended, and it will vary with the treating clinician. When counseling patients, it is important to acknowledge fatigue as an important and common symptom in PD, and not necessarily a reflection of depression. Many patients will have other potential contributors to fatigue, including depression, anxiety, apathy, sleep disorders and concomitant medical problems, and medications. These disorders are not always treatable. Although exercise has not been demonstrated in rigorous trials to reduce fatigue in PD, an interesting and repeated counterintuitive observation is that PD patients often report that they feel energized *after* exercising.^16,17^ As exercise is generally considered an important therapy for most aspects of PD and most medical disorders, we think exercise should always be suggested as there is no drawback.

## Current/future research

Current research on PD-related fatigue is focused on identification of biological markers and correlates of fatigue that may provide insights into the development of effective treatments. The Parkinson Progression Marker Initiative (PPMI) study (www.ppmi-info.org) is a biomarker-rich study focused on the course of early PD including ~425 newly diagnosed PD patients and ~200 matched health controls who undergo detailed assessments at baseline and annually (out to 5 years). Fatigue and its severity, as derived from the MDS-UPDRS fatigue item, will be evaluated for correlations with other measures including the following: serial dopamine transporter imaging, blood drawn for genotyping and assessment of plasma proteins, cerebrospinal fluid, serial magnetic resonance imaging brain imaging (structural and diffusion tensor imaging), and nonmotor phenomena, such as cognitive functioning, depression, anxiety, and daytime sleepiness.

Two additional ongoing studies on fatigue in PD personal communication) are using PET to examine neurophysiological correlates of fatigue. Preliminary results from FDG PET suggest a role for anterior cingulate, insular, superior temporal, and precuneus regions in PD fatigue (Strafella *et al.*, unpublished results). In a separate large PET study, the microglial ligand phenoxyanilide ([18[F]-FEPPA) is being used to examine the role of neuroinflammation in PD relative to fatigue severity based on the FSS.

Areas for future work include identification of which neurotransmitter systems or neural circuits, if any, are salient to the pathophysiology of fatigue in PD. Imaging of brain activity and other physiological measures will likely be utilized.

Comparing voxel-based morphometry data in PD patients with fatigue as well as older adults without fatigue may delineate brain structures associated with PD-related fatigue and whether these associations are similar to those seen in fatigue in older adults without PD.

Functional magnetic resonance imaging should be performed in fatigued and nonfatigued PD patients as free of confounding problems as possible (e.g., free of psychiatric or medical problems or medications associated with fatigue).

Several nonpharmacologic strategies have shown evidence for improving fatigue in MS but have not been tested in PD, including exercise, energy management strategies, and mindfulness-based stress reduction.^67^

### Conclusions

Further research is required to better understand fatigue in PD. The lack of progress in understanding the pathophysiology of fatigue, or its treatment, in more intensively studied disorders and the absence of any effective therapy for fatigue in any of them argues strongly for a greater effort. We believe that research to better understand the syndrome and treatment trials based on rational hypotheses of pathophysiology should proceed in parallel.

## Figures and Tables

**Table 1:** Published trials of treatments for fatigue

Modafinil
Methylphenidate
Pergolide
Bromocriptine
Pramipexole
Doxepin
Rasagaline
L-Dopa
Memantine
Caffeine
Behavioral interventions
Exercise
Amantadine
(from 60)

## Box

Fatigue: The patient perspective

## Box

*Anne Newbould (care partner)*: My husband of almost 40 years, James, has Parkinson's disease, and fatigue is his most severe and debilitating symptom. At first I thought he had depression. I attempted to get him interested in the things that we so loved to do, but it seemed that he lost his zeal for life. When I asked him about it, he told me that he was extremely tired and it almost hurt to try to get up out of bed or his chair. I started researching all I could on Parkinson's and fatigue, but there was very little information. I felt lost and unbelievably helpless. *We went from being participants to spectators in life*. With James' fatigue, we have had to alter our lifestyle to conform to his level of energy. We go to baseball games instead of fly-fishing. We cruise on the local Southern Bell riverboat instead of racing sailboats. We go to the beach and watch the ocean instead of teaching scuba diving. That is why James and I were ecstatic to have the opportunity to raise this issue through the Parkinson's Disease Foundation’s Community Choice Research Awards.

## Box

*Gracia Clark (patient)*: Fatigue was the first Parkinson's symptom I noticed; it is still my most debilitating one. Feeling fatigued is different from feeling sleepy, depressed or apathetic. As an anthropologist who has worked in West Africa, I am reminded of the fatigue I suffered after coming down with malaria. When fighting malaria, I faced an overwhelming need to lie down. But unlike fatigue from malaria, Parkinson’s fatigue doesn’t go away after taking a few malaria pills. Finding treatment for Parkinson’s fatigue can be a huge challenge. Medications approved for other diseases—such as narcolepsy—may provide some benefit, but their efficacy in Parkinson’s is under studied, and insurance companies can decline to cover such off-label uses. Furthermore, because many diseases can cause fatigue, receiving an accurate diagnosis can be difficult, as it was in my case. I have heard many people with Parkinson’s say fatigue is one of their most debilitating symptoms, and yet the symptom is not well understood. There is an urgent need for increased research to better understand fatigue and find ways to treat it.
